# Bacterial meningitis epidemiology and return of *Neisseria meningitidis* serogroup A cases in Burkina Faso in the five years following MenAfriVac mass vaccination campaign

**DOI:** 10.1371/journal.pone.0187466

**Published:** 2017-11-02

**Authors:** Alpha Oumar Diallo, Heidi M. Soeters, Issaka Yameogo, Guetawendé Sawadogo, Flavien Aké, Clément Lingani, Xin Wang, Andre Bita, Amadou Fall, Lassana Sangaré, Rasmata Ouédraogo-Traoré, Isaïe Medah, Brice Bicaba, Ryan T. Novak, for the MenAfriNet Consortium

**Affiliations:** 1 National Center for Immunization and Respiratory Diseases, Centers for Disease Control and Prevention, Atlanta, Georgia, United States of America; 2 Epidemic Intelligence Service, Centers for Disease Control and Prevention, Atlanta, Georgia, United States of America; 3 Direction de la Lutte contre la Maladie, Ministère de la Santé, Ouagadougou, Burkina Faso; 4 CDC Foundation, Ouagadougou, Burkina Faso; 5 World Health Organization, AFRO Intercountry Support Team for West Africa, Ouagadougou, Burkina Faso; 6 Centre Hospitalier Universitaire Yalgado Ouédraogo, Ouagadougou, Burkina Faso; 7 Centre Hospitalier Universitaire Pédiatrique Charles de Gaulle, Ouagadougou, Burkina Faso; Universidad Nacional de la Plata, ARGENTINA

## Abstract

**Background:**

Historically, *Neisseria meningitidis* serogroup A (NmA) caused large meningitis epidemics in sub-Saharan Africa. In 2010, Burkina Faso became the first country to implement a national meningococcal serogroup A conjugate vaccine (MACV) campaign. We analyzed nationwide meningitis surveillance data from Burkina Faso for the 5 years following MACV introduction.

**Methods:**

We examined Burkina Faso’s aggregate reporting and national laboratory-confirmed case-based meningitis surveillance data from 2011–2015. We calculated incidence (cases per 100,000 persons), and described reported NmA cases.

**Results:**

In 2011–2015, Burkina Faso reported 20,389 cases of suspected meningitis. A quarter (4,503) of suspected meningitis cases with cerebrospinal fluid specimens were laboratory-confirmed as either *S*. *pneumoniae* (57%), *N*. *meningitidis* (40%), or *H*. *influenzae* (2%). Average adjusted annual national incidence of meningococcal meningitis was 3.8 (range: 2.0–10.2 annually) and was highest among infants aged <1 year (8.4). *N*. *meningitidis* serogroup W caused the majority (64%) of meningococcal meningitis among all age groups. Only six confirmed NmA cases were reported in 2011–2015. Five cases were in children who were too young (n = 2) or otherwise not vaccinated (n = 3) during the 2010 MACV mass vaccination campaign; one case had documented MACV receipt, representing the first documented MACV failure.

**Conclusions:**

Meningococcal meningitis incidence in Burkina Faso remains relatively low following MACV introduction. However, a substantial burden remains and NmA transmission has persisted. MACV integration into routine childhood immunization programs is essential to ensure continued protection.

## Introduction

**Fig 1:**
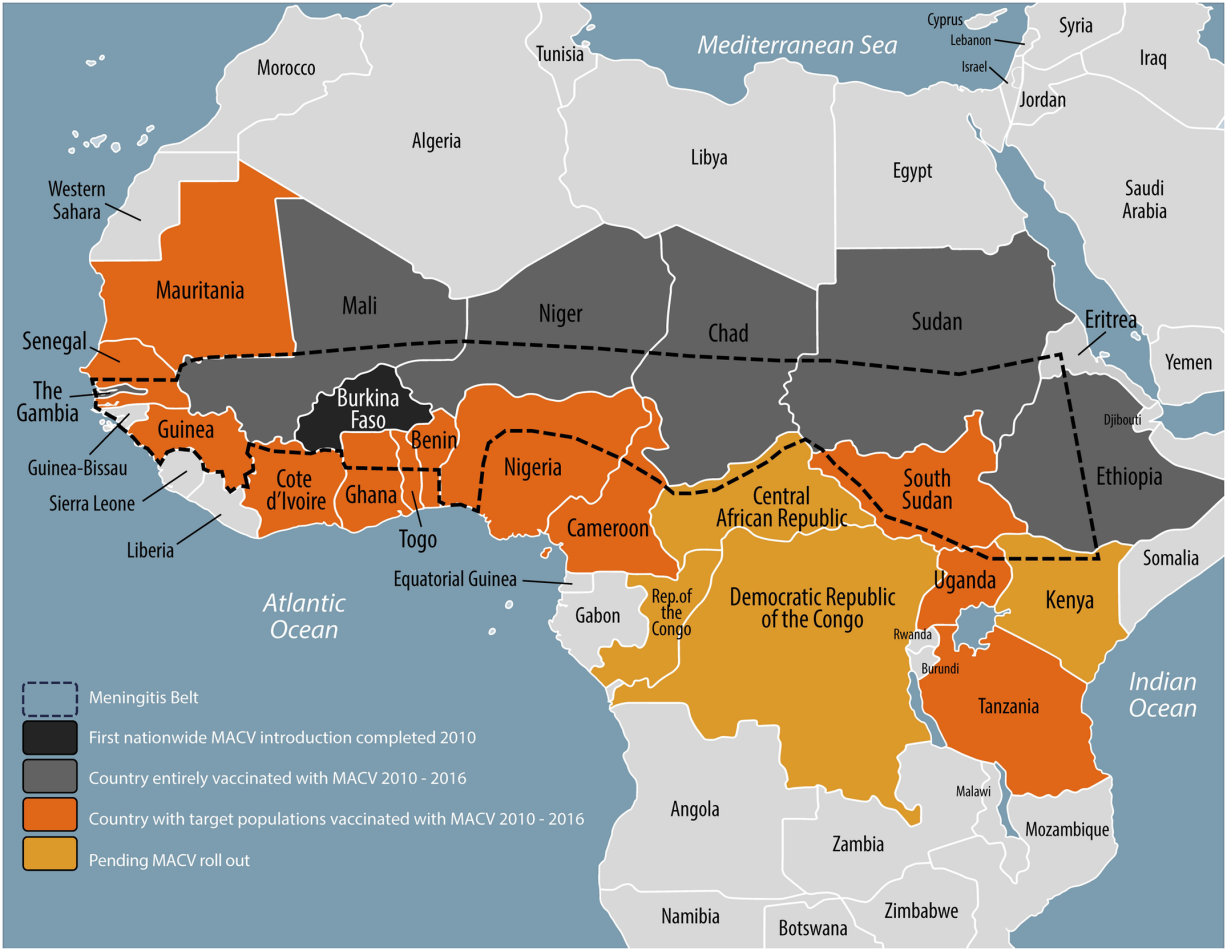
The meningitis belt of sub-Saharan Africa and meningococcal serogroup A conjugate vaccine (MACV) rollout, 2010–2016.

For over 100 years, the meningitis belt of sub-Saharan Africa—stretching from Senegal to Ethiopia and including 450 million people in 26 countries—experienced high endemic rates of meningitis, annual seasonal outbreaks, and explosive epidemics occurring every 5–12 years [1,2]. Prior to the introduction of the meningococcal serogroup A conjugate vaccine (MACV, MenAfriVac^™^), approximately 90% of meningitis cases during epidemics in the region were attributable to *Neisseria meningitidis* serogroup A (NmA) [3]. From 2010–2016, MACV was aggressively rolled out using national vaccination campaigns in 19 at-risk countries within or bordering the meningitis belt, representing a new approach to controlling epidemic-prone diseases (Fig 1) [4].

Burkina Faso, a landlocked West African country with a population of approximately 19 million, is one of the few countries entirely located within the meningitis belt and experiences hyper-endemic rates of meningitis [3]. In December 2010, Burkina Faso was the first country to complete national introduction of MACV through a 10-day mass vaccination campaign reaching 11 million people (approx. 70% of the population), achieving 96% coverage among the target population of persons aged 1–29 years [5]. An evaluation of the impact of MACV one-year following the campaign demonstrated a substantial reduction in meningitis incidence among both the target population and the general population, due to high coverage and herd protection [3].

A growing cohort of unvaccinated children (currently over 4 million) born since 2010 puts the population at risk of NmA epidemic resurgence. The World Health Organization (WHO) Strategic Advisory Group of Experts on Immunization’s 2014 recommendation of one dose of MACV at ≥9 months of age provides an opportunity to sustain the immunity already achieved [6]. Burkina Faso conducted a catch-up campaign among children aged 1–6 years in November 2016 and introduced a single dose of MACV into their routine immunization program in March 2017 at age 15–18 months, along with the second dose of measles-containing vaccine. We analyzed national meningitis surveillance data and described the epidemiology of reported NmA cases in the five years (2011–2015) since MACV introduction in Burkina Faso.

## Methods

### Meningitis surveillance

In Burkina Faso, two complementary systems of nationwide population-based meningitis surveillance exist [3]. Aggregate surveillance for reportable diseases is conducted via the *Télégramme Lettre Official Hebdomadaire*, which collects weekly reports of clinically-defined meningitis cases from both inpatient and outpatient facilities and meningitis-related deaths aggregated at the district-level. Functional since 1997, this system contains no identifying information or laboratory data, and only limited demographic information on age and sex. A second system, nationwide case-based meningitis surveillance, was implemented in 2010 prior to MACV introduction. This passive surveillance system collects case-level demographic and clinical information as well as results of cerebrospinal fluid (CSF) examination and laboratory testing using Integrated Disease Surveillance and Response tools [7]. A paper case notification form is completed for each suspected case, and district surveillance officers enter the data into a surveillance database. If a CSF specimen is collected, a copy of the case notification form travels with the specimen to district laboratories, regional laboratories, and national reference laboratories. At each level, laboratory results are entered into a laboratory database. Data are reported weekly to a central meningitis national reference laboratory and the Ministry of Health surveillance office, including zero reporting. The surveillance office merges the surveillance database with the laboratory database to create a master database, which includes cases both with and without CSF specimens and laboratory results. Burkina Faso, as a member of the MenAfriNet Consortium (www.menafrinet.org), makes substantial efforts to maintain high-quality cased-based surveillance with quarterly assessments of performance indicators covering critical surveillance domains, including specimen collection and transport to a national reference laboratory, pathogen confirmation, linkage of laboratory and epidemiologic data, and data management.

### Case definitions

Cases are classified according to WHO case definitions [8]. A case of suspected meningitis is defined as sudden onset of fever ≥38.5°C with one of the following signs: neck stiffness, altered consciousness, or other meningeal signs (including flaccid neck, bulging fontanel, or convulsions in young children). Probable bacterial meningitis is a suspected case with turbid, cloudy, purulent, or xanthochromic CSF; presence of Gram negative diplococci, Gram positive diplococci, or Gram negative bacilli on microscopic examination of CSF; or a CSF white cell count >10/mm^3^. A confirmed case of meningitis is a suspected or probable case with *N*. *meningitidis*, *Streptococcus pneumoniae*, or *Haemophilus influenzae* isolated from CSF by culture or detected in CSF by real-time polymerase chain reaction (rt-PCR) or latex agglutination [9].

### Laboratory methods

CSF specimens are transported from local healthcare facilities to district laboratories, which conduct preliminary lab testing such as cytology, Gram staining, and latex agglutination (Pastorex, Bio-Rad). CSF specimens are also sent to a national reference laboratory for culture and rt-PCR targeting the *sodC* gene for *N*. *meningitidis*, *lytA* for *S*. *pneumoniae*, and *hpd* for *H*. *influenzae* [10, 11]. Meningococcal serogroups are determined using latex agglutination or rt-PCR, with rt-PCR considered definitive [12]; sequence type and clonal complex were determined using whole genome sequencing.

### NmA case investigations

Each reported NmA case in Burkina Faso triggers a full case investigation, including an initial investigation by district health officers and a follow-up investigation by a national team of epidemiologists and laboratory technicians. The investigations confirm the patient’s age, sex, vaccination status, travel history, epidemiologic links, and the causative agents confirmed by laboratory tests.

### Statistical analysis

To assess the impact of MACV on meningitis epidemiology in the 5 years following MACV introduction in 2010, we examined both aggregate and case-based meningitis surveillance data from January 1, 2011, to December 31, 2015. Cases among non-residents of Burkina Faso were excluded. Incidence rates (cases per 100,000 persons) were calculated using national census estimates. District-level epidemics were defined by an aggregate suspected meningitis incidence exceeding 100 per 100,000 population per week. The sensitivity of the case-based surveillance system to detect suspected meningitis cases was calculated using aggregate surveillance case counts as the denominator.

For laboratory-confirmed cases, annual incidence was adjusted for the age-stratified proportion of cases with CSF tested at a national laboratory, where culture and rt-PCR were performed. Within each age stratum (<1 year, 1–4 years, 5–9 years, 10–14 years, 15–29 years and ≥30 years), the number of cases confirmed by culture or rt-PCR for a specific pathogen was divided by the number of cases with CSF tested via culture or rt-PCR at a national laboratory; this proportion was then applied to cases lacking any test results within that age stratum.

Data were analyzed using SAS v9.3. This evaluation was determined by the Centers for Disease Control and Prevention’s Human Research Protection Office to be public health non-research, and Institutional Review Board review was not required.

## Results

### Aggregate meningitis surveillance

From 2011–2015, 20,389 cases of suspected meningitis and 2,333 (12%) deaths were reported via aggregate surveillance in Burkina Faso, corresponding to an annual median of 3,486 cases (range: 2,919–7,022) and average annual incidence of 24.5 cases per 100,000 population (Tables 1 and 2). Overall, 15,629 (77%) of cases occurred during the meningitis season (epidemiologic weeks 1–24). A total of 7 district-level epidemics occurred, all in 2012.

**Table 1:** Aggregate and case-based meningitis surveillance data indicators, Burkina Faso, 2011–2015.

	2011	2012	2013	2014	2015	Total
	N (%)					
***Aggregate meningitis surveillance***						
Suspected meningitis cases	3,878	7,022	2,919	3,486	3,084	20,389
District-level epidemics*	0	7	0	0	0	7
Deaths	588 (15)	739 (11)	339 (12)	357 (10)	310 (10)	2,333 (11)
***Case-based meningitis surveillance***						
Districts reporting	57/63 (90)	63/63 (100)	63/63 (100)	63/63 (100)	63/63 (100)	63/63 (100)
Suspected case-based meningitis cases	2,841	6,499	2,829	3,399	2,970	18,538
Cases with CSF specimen†	2,767 (97)	6,302 (97)	2,758 (97)	3,331 (98)	2,912 (98)	18,069 (97)
CSFs with a Gram stain	2,508 (91)	5,626 (89)	2,460 (89)	2,949 (89)	2,686 (92)	16,229 (90)
CSFs tested by latex, culture or rt-PCR	1,718 (62)	2,894 (46)	2,011 (73)	2,312 (69)	2,229 (77)	11,013 (64)
CSFs tested by latex	1,146 (41)	1,205 (19)	513 (19)	716 (22)	308 (11)	3,888 (22)
CSFs tested by culture	722 (26)	1,552 (25)	637 (23)	563 (17)	420 (14)	3,894 (22)
CSFs cultured but found to be contaminated	190 (26)	275 (18)	107 (17)	123 (22)	98 (23)	793 (20)
CSFs tested by rt-PCR	1,133 (41)	1,396 (22)	1,704 (62)	1,958 (59)	2,115 (73)	8,306 (46)
CSFs confirmed for Hi/Nm/Sp	860 (31)	1,293 (21)	705 (26)	762 (23)	883 (30)	4,503 (25)

Abbreviations: CSF, cerebrospinal fluid; Hi, *Haemophilus influenzae*; Nm, *Neisseria meningitidis*; rt-PCR, real-time polymerase chain reaction; Sp, *Streptococcus pneumoniae*.

*A district-level epidemic was defined as an aggregate suspected meningitis incidence rate exceeding 100 per 100,000 population per week.

^†^The number of cases with a CSF specimen collected serves as the denominator for all below categories.

**Table 2:** Annual incidence (cases per 100,000 persons) of suspected, probable and laboratory-confirmed meningitis, Burkina Faso, 2011–2015.

	2011	2012	2013	2014	2015	Average annual incidence
***Aggregate meningitis surveillance***						
Suspected meningitis cases*	25.9	41.8	16.9	20.1	16.9	24.3
***Case-based meningitis surveillance***						
Suspected meningitis cases*	19.0	38.7	16.3	19.6	17.1	22.2
<1 year	94.6	176.3	81.5	88.3	65.8	101.3
1–4 years	30.0	77.9	31.9	37.4	32.3	41.9
5–9 years	21.2	50.0	18.9	22.0	20.1	26.4
10–14 years	20.4	37.1	14.4	17.9	15.9	21.1
15–29 years	9.5	15.1	7.6	8.8	7.7	9.7
≥30 years	7.7	11.6	5.3	7.0	5.7	7.4
Probable meningitis cases*	10.0	22.2	7.9	9.0	8.6	13.4
Laboratory-confirmed meningitis cases†‡	9.4	16.7	5.7	6.0	6.8	10.0
*N*. *meningitidis*	2.1	10.2	2.1	2.0	2.3	3.7
Serogroup						
A	0.0	0	0	0.0	0.0	0.0
C	0	0	0	0	0.1	0.0
W	0.6	8.7	1.9	1.9	1.9	3.0
X	1.5	1.4	0.2	0	0.1	0.6
Y	0	0.0	0	0	0.0	0.0
Age group						
<1 year	3.5	24.4	4.6	5.0	4.3	8.4
1–4 years	3.3	19.4	3.3	3.3	3.9	6.6
5–9 years	3.9	18.6	3.9	3.3	4.1	6.8
10–14 years	4.4	13.3	2.8	2.6	2.7	5.2
15–29 years	0.6	3.9	1.3	0.9	0.8	1.5
≥30 years	0.3	1.8	0.4	0.5	0.7	0.8
*S*. *pneumoniae*	7.1	6.2	3.5	3.8	3.9	4.9
*H*. *influenzae*	0.3	0.2	0.1	0.1	0.2	0.2

*Crude incidence.

^†^ Confirmed via latex agglutination, culture, or real-time polymerase chain reaction as *N*. *meningitidis*, *S*. *pneumoniae*, or *H*. *influenzae*.

^‡^Incidence adjusted for age and the proportion of cases with cerebrospinal fluid tested at a national laboratory. An incidence of 0 indicates no confirmed cases with that specific pathogen in that year, whereas an incidence of 0.0 indicates that cases with that pathogen occurred, but at a low incidence rounding to 0.0.

### Case-based meningitis surveillance

**Fig 2:**
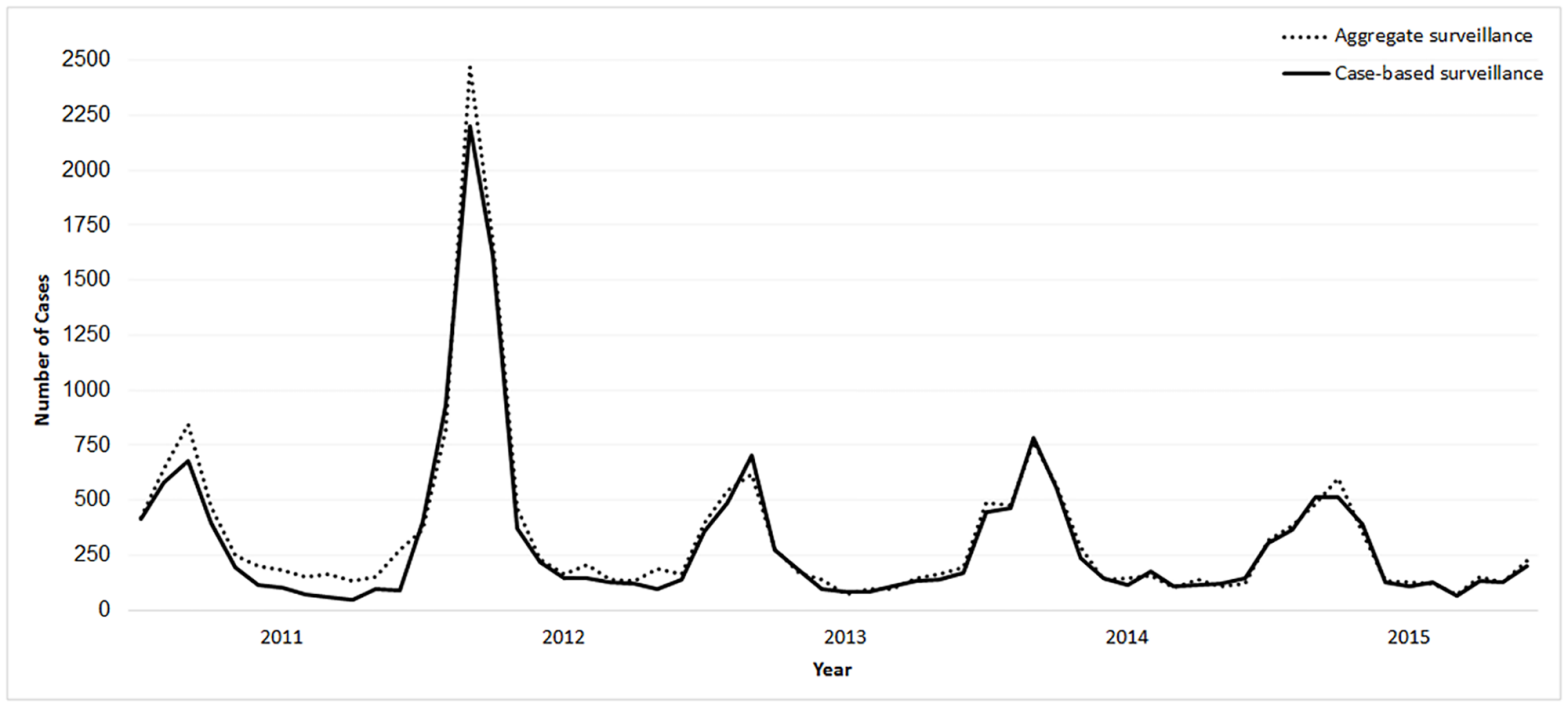
Epidemic curve of suspected meningitis cases reported weekly to aggregate and case-based meningitis surveillance systems, Burkina Faso, 2011–2015.

During 2011–2015, 18,538 individual suspected meningitis cases and 1,996 (11%) deaths were reported through case-based meningitis surveillance (Tables 1 and 3). The numbers of cases reported per week by both surveillance systems were similar, except in 2011 and 2015, when some reporting lags in the aggregate surveillance system were apparent (Fig 2). The annual sensitivity of the case-based surveillance system to detect suspected meningitis cases, using aggregate surveillance for comparison, improved from 73% (2,842/3,878) in 2011 to 96% (2,970/3,084) in 2015.

**Table 3:** Suspected, probable and laboratory-confirmed meningitis cases from case-based meningitis surveillance, Burkina Faso, 2011–2015.

	2011	2012	2013	2014	2015	Total
	N (%)					
Suspected meningitis cases	2,841	6,499	2,829	3,399	2,970	18,538
Age group*						
<1 years	591 (21)	1,219 (19)	574 (20)	635 (19)	482 (16)	3,501 (19)
1–4 years	667 (24)	1,938 (30)	810 (29)	970 (29)	856 (29)	5,241 (28)
5–9 years	504 (18)	1,323 (20)	516 (18)	620 (18)	585 (20)	3,548 (19)
10–14 years	402 (14)	826 (13)	333 (12)	430 (13)	395 (13)	2,386 (13)
15–29 years	366 (13)	655 (10)	341 (12)	414 (12)	375 (13)	2,151 (12)
≥30 years	298 (11)	508 (8)	240 (9)	325 (10)	272 (9)	1,643 (9)
Sex**						
Female	1,298 (46)	2,851 (44)	1,222 (43)	1,533 (45)	1,328 (45)	8,232 (44)
Male	1,536 (54)	3,634 (56)	1,597 (57)	1,865 (55)	1,642 (55)	10,274 (56)
Reported deaths	419 (15)	590 (9)	335 (12)	349 (10)	303 (10)	1,996 (11)
Probable bacterial meningitis cases†	1,488 (54)	3,727 (59)	1,377 (50)	1,564 (47)	1,483 (51)	9,639 (53)
Laboratory-confirmed meningitis cases‡	860 (31)	1,293 (21)	705 (26)	762 (23)	883 (30)	4,503 (25)
*N*. *meningitidis*	192 (22)	813 (63)	264 (37)	240 (32)	302 (34)	1,811 (40)
Serogroup						
A	1 (1)	0 (0)	0 (0)	1 (0)	4 (1)	6 (0)
C	0 (0)	0 (0)	0 (0)	0 (0)	10 (3)	10 (1)
W	34 (18)	504 (62)	213 (81)	149 (62)	258 (85)	1,158 (64)
X	99 (52)	82 (10)	23 (8)	2 (1)	6 (2)	212 (12)
Y	0 (0)	2 (0)	0 (0)	0 (0)	4 (1)	6 (0)
Nongroupable	58 (30)	225 (28)	28 (11)	88 (37)	20 (7)	419 (23)
Reported deaths	21 (11)	62 (8)	28 (11)	23 (10)	19 (6)	153 (8)
*S*. *pneumoniae*	642 (75)	462 (36)	424 (60)	502 (66)	551 (62)	2,581 (57)
*H*. *influenzae*	26 (3)	18 (1)	17 (2)	20 (3)	30 (3)	111 (2)
Serotype b	26 (100)	18 (100)	17 (100)	18 (90)	19 (63)	98 (88)
Non-b serotypes	0 (0)	0 (0)	0 (0)	2 (10)	11 (37)	13 (12)

*68 cases missing age

**32 cases missing sex

^†^Probable bacterial meningitis is a suspected case with turbid, cloudy, purulent, or xanthochromic cerebrospinal fluid (CSF); or presence of Gram negative diplococci, Gram positive diplococci, or Gram negative bacilli on microscopic examination of CSF; or a CSF white cell count >10/mm^3^.

^‡^Confirmed via latex agglutination, culture, or real-time polymerase chain reaction as *N*. *meningitidis*, *S*. *pneumoniae*, or *H*. *influenzae*.

### Data completeness

Of the 63 districts in Burkina Faso, 57 (90%) submitted case-based surveillance data in 2011 and 63 (100%) in 2012–2015 (Table 1). A consistently high proportion (97%) of reported cases received lumbar punctures. Ninety percent of suspected cases had a CSF specimen tested by Gram stain. Sixty-four percent of specimens were tested by at least one confirmatory test: 22% by latex, 22% by culture, and 46% by rt-PCR.

### Suspected and probable meningitis cases

Nearly a fifth (19%) of suspected meningitis cases occurred among children aged <1 year and 47% occurred in children aged <5 years (Table 3). Fifty-three percent (n = 9,639) of all suspected cases met the probable case definition. The annual average incidences of suspected and probable meningitis were 22.2 and 13.4 cases per 100,000, respectively (Table 2).

### Laboratory-confirmed meningitis

**Fig 3:**
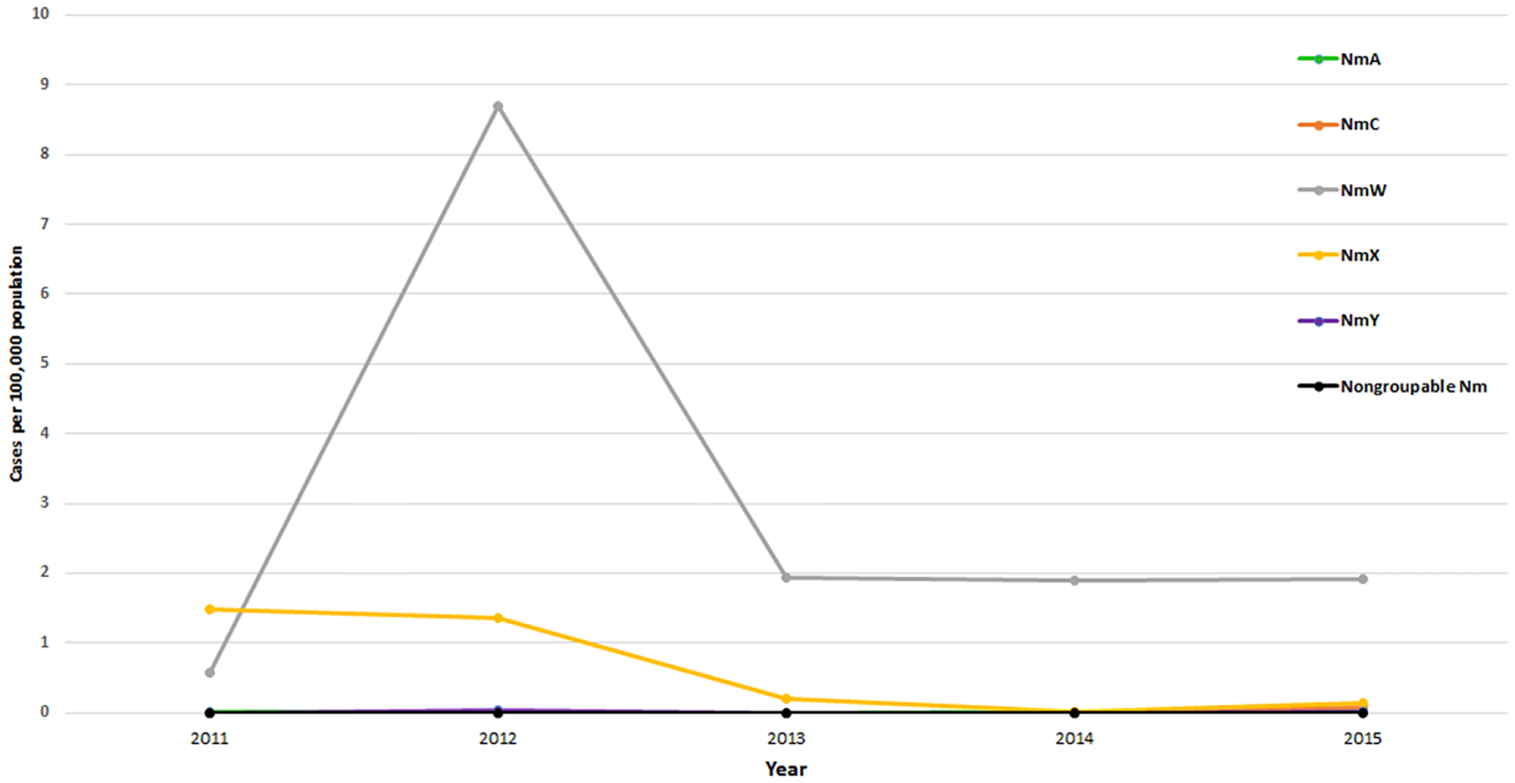
Adjusted annual incidence of meningococcal meningitis by serogroup, Burkina Faso, 2011–2015. Abbreviations: Nm, *N*. *meningitidis*; NmA, *N*. *meningitidis* serogroup A; NmC, *N*. *meningitidis* serogroup C; NmW, *N*. *meningitidis* serogroup W; NmX, *N*. *meningitidis* serogroup X; NmY, *N*. *meningitidis* serogroup Y.

A quarter (n = 4,503) of suspected cases were laboratory-confirmed via latex, culture or rt-PCR as either *N*. *meningitidis* (40%), *S*. *pneumoniae* (57%), or *H*. *influenza*e (2%) (Table 2). *S*. *pneumoniae* was the predominant pathogen (average annual adjusted incidence: 5.0), except in 2012, when there was a serogroup W meningococcal (NmW) meningitis epidemic and NmW accounted for 65% of laboratory-confirmed meningitis cases (Fig 3).

**Fig 4:**
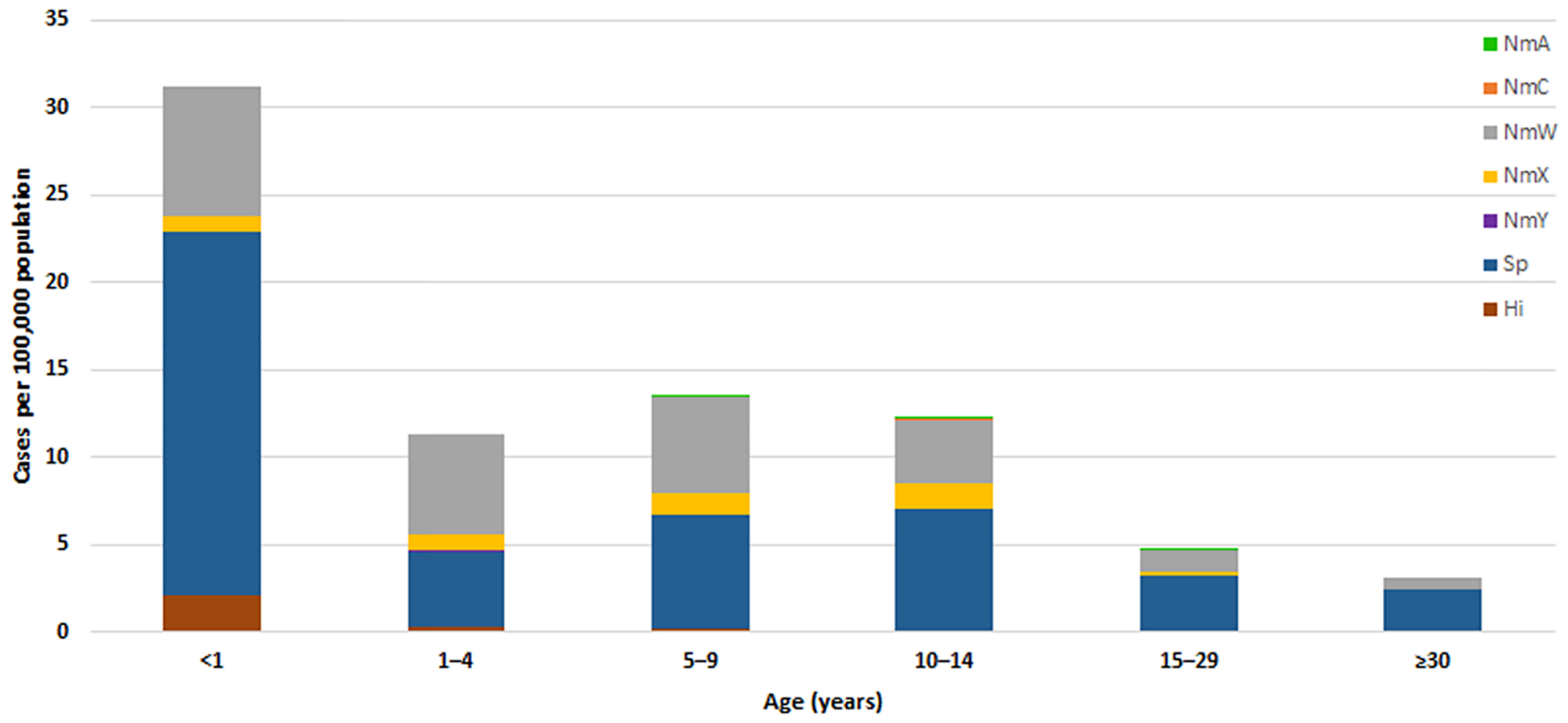
Average adjusted annual incidence of laboratory-confirmed meningitis by pathogen and age group, Burkina Faso, 2011–2015. Abbreviations: Hi, *H*. *influenzae*; NmA, *N*. *meningitidis* serogroup A; NmC, *N*. *meningitidis* serogroup C; NmW, *N*. *meningitidis* serogroup W; NmX, *N*. *meningitidis* serogroup X; NmY, *N*. *meningitidis* serogroup Y; Sp, *S*. *pneumoniae*.

Overall, 1,811 meningococcal meningitis cases were reported in 2011–2015 (Table 2); 153 (8%) were fatal. Apart from the elevated incidence of *N*. *meningitidis* in 2012 (10.6 per 100,000), the annual incidence remained low (range: 1.7–2.1), with an average annual incidence of 3.8 in 2011–2015 (Table 2). One-third (34%) of meningococcal meningitis cases occurred among children aged <5 years (incidence: 15.0 per 100,000), with the highest incidence among infants aged <1 year (8.4 per 100,000) (Table 2, Fig 4, S1 Table). NmW accounted for the highest incidence among meningococcal serogroups (Table 2, Fig 3). The incidence of serogroup X (NmX) declined over time (1.5 in 2011 to 0.1 in 2015); however, cases were widely distributed nationwide (46 of 63 districts had at least one NmX case in 2011–2015). After four consecutive years without any reported serogroup C (NmC) cases, NmC reemerged in 2015 (incidence: 0.1), with 10 cases occurring in 6 districts, primarily in northern Burkina Faso.

### NmA cases

From 2011–2015, six confirmed NmA cases were reported in Burkina Faso: 1 in 2011, 1 in 2014, and 4 in 2015 (Table 4), resulting in an average annual adjusted incidence of 0.01 per 100,000. All NmA cases were confirmed using rt-PCR; and the 2011 case was additionally confirmed using latex agglutination and culture. Five of 6 cases occurred between November 2014 and May 2015 in three adjacent districts in northern Burkina Faso: Ouahigouya, Titao and Tougan.

**Table 4:** Reported *N*. *meningitidis* serogroup A cases, Burkina Faso, 2011–2015.

Year	Epidemiologic Week	Region	District	Age (years)	Sex	Vaccinated with MACV	Outcome	Gram stain	Latex agglutination	Culture	rt-PCR	Sequence type	Clonal complex
2011	8	Cascades	Banfora	17	F	No	Survived	DGN	NmA	NmA	NmA	NP^†^	NP^†^
2014	48	Nord	Ouahigouya	19	M	No	Survived*	DGN	NP	NP	NmA	7	ST-5 complex/subgroup III
2015	12	Nord	Ouahigouya	5	F	No	Survived	DGN	NP	NP	NmA	7	ST-5 complex/subgroup III
2015	18	Mouhoun	Tougan	12	M	No	Survived	DGN	NP	NP	NmA	7	ST-5 complex/subgroup III
2015	18	Nord	Ouahigouya	9	F	Yes	Survived	DGN	NP	NP	NmA	NP^‡^	NP^‡^
2015	22	Nord	Titao	5	M	No	Died	DGN	NP	NP	NmA	7	ST-5 complex/subgroup III

Abbreviations: DGN, Gram-negative diplococcus; F, female; M, male; MACV, meningococcal serogroup A conjugate vaccine; NmA, *N*. *meningitidis* serogroup A; NP, not performed; rt-PCR, real-time polymerase chain reaction.

*Hearing loss.

^†^The specimen was no longer available for sequencing.

^‡^The remaining cerebrospinal fluid quantity was insufficient for sequencing.

The NmA patients ranged in age from 5–19 years; half were female (Table 4). None traveled outside their community in the month before disease onset. No epidemiologic links between cases were identified. One case was fatal and one resulted in hearing loss.

Two of the six NmA cases occurred in 5-year-old children who were too young to be vaccinated during the 2010 MACV mass campaign, and three occurred in children who were age-eligible for MACV in 2010 but were not vaccinated. The remaining case occurred in a 9-year-old female who, based on her vaccination card, received MACV 5 years earlier (during the mass vaccination campaign); this represents the first documented case of MACV failure. Serum specimens were not available for complement component deficiency or immunologic testing.

## Discussion

This analysis of bacterial meningitis epidemiology was based on aggregate and case-based meningitis surveillance systems in Burkina Faso, a country which maintained high-quality surveillance in the five years post-MACV. These data provide evidence that MACV introduction resulted in a continued lower burden of suspected meningitis (aggregate reporting average incidence: 24.3 cases per 100,000 population) compared to the 4 years prior to MACV introduction (aggregate reporting average incidence 81.2 cases per 100,000) [3]. The highest incidence of suspected meningitis was observed among children aged <5 years, who were too young to be vaccinated during or were born after the 2010 MACV national campaign.

These results support previous findings demonstrating the impact of MACV in Burkina Faso and elsewhere in the meningitis belt. Remarkable decreases in meningococcal disease incidence, particularly for NmA disease, have been shown in other countries that introduced MACV [13]. Cross-sectional meningococcal carriage surveys conducted before and a year after MACV introduction demonstrated elimination of NmA carriage among both vaccinated and unvaccinated populations in Burkina Faso [14]. Similar results have been observed elsewhere in the meningitis belt, implying that there is a vaccine-induced herd protection effect [15, 16]. Additionally, it has been shown that a single dose of MACV induces sustained levels of NmA antibodies in children aged 12–23 months for up to five years [17]. These findings, along with the high community acceptance [18] and low cost of the vaccine, provide compelling evidence for continued MACV rollout in all countries inside or bordering the meningitis belt [4, 19, 20].

Of six NmA cases detected among Burkina Faso residents since MACV introduction in 2010, five occurred during the 2014–2015 meningitis epidemic season, with two of these occurring in children who were too young to be vaccinated during the 2010 MACV campaign. While these cases were reported and thoroughly investigated, it is possible that additional NmA cases occurred but were undetected, unconfirmed, or unreported. Confirmation of the six cases described here suggests a recent increase in NmA transmission. This is likely the result of an increase in the pool of susceptible persons and a decrease in herd protection that occurred as years passed without routine MACV infant vaccination or catch-up campaigns for children born after the mass MACV campaign. In 2015, the estimated susceptible population eligible for MACV vaccination in Burkina Faso was over 3.5 million, equaling at least 30% of the population size originally vaccinated with MACV in 2010. To assess the long-term impact of MACV on the prevalence, serogroup distribution, and molecular characteristics of nasopharyngeal carriage of *N*. *meningitidis*, particularly NmA, meningococcal carriage evaluations are underway in Burkina Faso in 2016–2017. These evaluations include the districts of Ouahigouya, where three of the five recent NmA cases were reported, and Kaya, which had the highest NmA carriage prevalence prior to MACV introduction [14].

Burkina Faso’s detection, investigation, confirmation, and reporting of NmA cases motivated Gavi and the international community to accelerate Burkina Faso’s timeline for MACV introduction into the Expanded Programme on Immunization (EPI), in order to halt NmA transmission. Burkina Faso conducted a MACV catch-up campaign of infants and children aged 1–5 years in November 2016 and integrated MACV into the EPI in March 2017 for administration to children aged 15–18 months. Mathematical models have shown that the strategy implemented in Burkina Faso—conducting catch-up campaigns prior to EPI introduction—would produce the lowest overall annual incidence of NmA meningitis and maintain long-term population protection [21, 22]. The high community acceptance of MACV may also benefit other EPI vaccines co-administered at the same age [18, 23]. Burkina Faso strategically decided to integrate MACV into their EPI at age 15–18 months concomitantly with the second dose of measles-containing vaccine (MCV2); a follow-up MACV and MCV2 vaccination coverage survey is planned to measure potential impact of MACV EPI introduction on MCV2 vaccination coverage.

Burkina Faso’s NmA case investigations confirmed that one of the six patients was vaccinated with MACV in 2010, signifying the first reported MACV failure among the estimated 260 million individuals vaccinated across 19 countries in the meningitis belt since 2010.^4^ The country’s investigation and transparent reporting to the international community are a key component of monitoring MACV implementation and success. The MenAfriNet Consortium developed a Serogroup A Case Investigation Protocol based on Burkina Faso’s experience, to guide standardized data collection, laboratory confirmation, and reporting for each NmA case detected in all countries that have introduced MACV, regardless of surveillance capacity.

In addition to the reemergence of NmA disease in Burkina Faso and other countries that previously introduced MACV (Benin, Ghana, Guinea, Mali and Togo) [24], regional meningitis epidemiology is changing. *S*. *pneumoniae* has been the predominant endemic bacterial meningitis pathogen in Burkina Faso since 2011 [25], and *N*. *meningitidis* serogroup W has accounted for the majority of epidemic meningitis [26]. Regionally, serogroup C and X have also emerged as significant causes of epidemic meningitis: serogroup C tends to be clustered, while serogroup X has been dispersed. The strain identified in the serogroup C case reported by Burkina Faso in 2015 shared similar molecular characteristics with the serogroup C strain that emerged in Nigeria in 2013 and caused the largest global epidemic of this serogroup in Niger in 2015 [27–29]. Burkina Faso’s experience in conducting high-quality meningitis surveillance demonstrates that long-term investment in case-based surveillance is a valuable platform both for monitoring evolving meningitis epidemiology and for evaluating the effectiveness of bacterial meningitis vaccines, especially MACV integration into the EPI, pneumococcal conjugate vaccines, and a potential polyvalent meningococcal conjugate vaccine [30]. Despite increases in surveillance quality over time in Burkina Faso, challenges remain regarding specimen transport and laboratory confirmation (see S2 Table); however reported incidence rates adjust for changes in culture and real-time PCR testing capacity over time and likely reflect true trends in pathogen-specific incidences.

An immense effort has been invested in mass vaccination campaigns in Burkina Faso and other countries in the region, resulting in major successes in mobilizing international and local communities and encouraging high vaccine uptake, and in an extraordinary and unprecedented reduction in meningitis burden and NmA transmission. However, the data provide concerning evidence of a return of NmA transmission and disease as the susceptible population size increases. It is critical that the initial MACV roll-out is completed in the remaining countries of the meningitis belt and that MACV is thoughtfully and successfully integrated into EPI programs [30]. Continuing the momentum of MACV roll-out, along with long-term investments in surveillance and thorough investigations into reported NmA cases, must remain a high priority for the international community and countries within the meningitis belt. Failure to take these measures will undermine the outstanding successes that have already been achieved, and will place future generations at risk of experiencing devastating meningitis epidemics.

## Supporting information

S1 File Dataset. (XLSX)

S1 Table Annual incidence (cases per 100,000 persons) of meningococcal meningitis by age group and serogroup, Burkina Faso, 2011–2015. (DOCX)

S2 Table Comparison of suspected meningitis cases tested vs. not tested at a national reference laboratory, Burkina Faso, 2011–2015. (DOCX)
