# Supplementary material for: Bacterial meningitis epidemiology and return of Neisseria meningitidis serogroup A cases in Burkina Faso in the five years following MenAfriVac mass vaccination campaign
Source: PLoS One. 2017 Nov 2;12(11):e0187466. doi: 10.1371/journal.pone.0187466 (PMC5667755; doi:10.1371/journal.pone.0187466)
Supplement: S1 Table — (DOCX) [file pone.0187466.s002.docx]

**S1 Table.** **Annual incidence (cases per 100,000 persons) of meningococcal meningitis by age group and serogroup, Burkina Faso, 2011–2015**

|  | 2011 | 2012 | 2013 | 2014 | 2015 | Average annual incidence |
| --- | --- | --- | --- | --- | --- | --- |
| Meningococcal meningitis*† |  |  |  |  |  |  |
| <1 year | 3.5 | 24.4 | 4.6 | 5.0 | 4.3 | 8.4 |
| NmA | 0 | 0 | 0 | 0 | 0 | 0 |
| NmC | 0 | 0 | 0 | 0 | 0 | 0 |
| NmW | 1.3 | 23.0 | 3.8 | 4.6 | 4.3 | 7.4 |
| NmX | 2.2 | 1.0 | 0.7 | 0.5 | 0 | 0.9 |
| NmY | 0 | 0.5 | 0 | 0 | 0 | 0 |
| 1–4 years | 3.3 | 19.4 | 3.3 | 3.3 | 3.9 | 6.6 |
| NmA | 0 | 0 | 0 | 0 | 0 | 0 |
| NmC | 0 | 0 | 0 | 0 | 0 | 0 |
| NmW | 1.3 | 17.0 | 3.2 | 3.3 | 3.7 | 5.7 |
| NmX | 2.0 | 2.4 | 0.1 | 0 | 0.1 | 0.9 |
| NmY | 0 | 0 | 0 | 0 | 0.2 | 0.0 |
| 5–9 years | 3.9 | 18.6 | 3.9 | 3.3 | 4.1 | 6.8 |
| NmA | 0 | 0 | 0 | 0 | 0.1 | 0.0 |
| NmC | 0 | 0 | 0 | 0 | 0.2 | 0.0 |
| NmW | 0.9 | 15.7 | 3.7 | 3.3 | 3.7 | 5.4 |
| NmX | 3.0 | 2.9 | 0.2 | 0 | 0.1 | 1.2 |
| NmY | 0 | 0.1 | 0 | 0 | 0 | 0.0 |
| 10–14 years | 4.4 | 13.3 | 2.8 | 2.6 | 2.7 | 5.2 |
| NmA | 0 | 0 | 0 | 0 | 0.1 | 0.0 |
| NmC | 0 | 0 | 0 | 0 | 0.3 | 0.1 |
| NmW | 0.6 | 10.5 | 2.1 | 2.6 | 2.3 | 3.6 |
| NmX | 3.8 | 2.9 | 0.7 | 0 | 0.1 | 1.5 |
| NmY | 0 | 0 | 0 | 0 | 0 | 0 |
| 15–29 years | 0.6 | 3.9 | 1.3 | 0.9 | 0.8 | 1.5 |
| NmA | 0.1 | 0 | 0 | 0.1 | 0 | 0.0 |
| NmC | 0 | 0 | 0 | 0 | 0 | 0 |
| NmW | 0.2 | 3.5 | 1.2 | 0.8 | 0.4 | 1.2 |
| NmX | 0.2 | 0.4 | 0.1 | 0 | 0.4 | 0.2 |
| NmY | 0 | 0 | 0 | 0 | 0 | 0 |
| ≥30 years | 0.3 | 1.8 | 0.4 | 0.5 | 0.7 | 0.8 |
| NmA | 0 | 0 | 0 | 0 | 0 | 0 |
| NmC | 0 | 0 | 0 | 0 | 0 | 0 |
| NmW | 0.2 | 1.6 | 0.4 | 0.5 | 0.7 | 0.7 |
| NmX | 0.2 | 0.1 | 0.0 | 0 | 0 | 0.1 |
| NmY | 0 | 0 | 0 | 0 | 0 | 0 |

Abbreviations: NmA, *N. meningitidis* serogroup A; NmC, *N. meningitidis* serogroup C; NmW, *N. meningitidis* serogroup W; NmX, *N. meningitidis* serogroup X; NmY, *N. meningitidis* serogroup Y.

* Confirmed via latex agglutination, culture, or real-time polymerase chain reaction as *N. meningitidis.*

† Incidence adjusted for the proportion of cases with cerebrospinal fluid tested at a national laboratory. An incidence of 0 indicates no confirmed cases with that specific pathogen in that year, whereas an incidence of 0.0 indicates that cases with that pathogen occurred, but at a low incidence rounding to 0.0.
