# Supplementary material for: Bacterial meningitis epidemiology and return of Neisseria meningitidis serogroup A cases in Burkina Faso in the five years following MenAfriVac mass vaccination campaign
Source: PLoS One. 2017 Nov 2;12(11):e0187466. doi: 10.1371/journal.pone.0187466 (PMC5667755; doi:10.1371/journal.pone.0187466)
Supplement: S2 Table — (DOCX) [file pone.0187466.s003.docx]

**S2 Table. Comparison of suspected meningitis cases tested^a^ vs. not tested at a national reference laboratory, Burkina Faso, 2011–2015**

|  | 2011 | | 2012 | | 2013 | | 2014 | | 2015 | | Total | |
| --- | --- | --- | --- | --- | --- | --- | --- | --- | --- | --- | --- | --- |
|  | Tested | Not Tested | Tested | Not Tested | Tested | Not Tested | Tested | Not Tested | Tested | Not Tested | Tested | Not Tested |
|  | N (%) | | | | | | | | | | | |
| Suspected cases^b^ | 1,242 (44) | 1,599 (56) | 2,379 (37) | 4,120 (63) | 1,836 (65) | 993 (35) | 2,039 (60) | 1,360 (40) | 2,166 (73) | 804 (27) | 9,662 (52) | 8,876 (48) |
| Age group^c^ |  |  |  |  |  |  |  |  |  |  |  |  |
| <1 years | 240 (19) | 351 (22) | 408 (17) | 811 (20) | 384 (21) | 190 (19) | 392 (19) | 243 (18) | 337 (16) | 145 (18) | 1,761 (18) | 1,740 (20) |
| 1–4 years | 234 (19) | 433 (27) | 679 (29) | 1,259 (31) | 518 (28) | 292 (30) | 549 (27) | 421 (31) | 621 (29) | 235 (29) | 2,601 (27) | 2,640 (30) |
| 5–9 years | 237 (19) | 267 (17) | 515 (22) | 808 (20) | 357 (19) | 159 (16) | 383 (19) | 237 (17) | 446 (21) | 139 (17) | 1,938 (20) | 1,610 (18) |
| 10–14 years | 202 (16) | 200 (13) | 345 (15) | 481 (12) | 223 (12) | 110 (11) | 254 (12) | 176 (13) | 276 (13) | 119 (15) | 1,300 (13) | 1,086 (12) |
| 15–29 years | 182 (15) | 184 (12) | 223 (9) | 432 (11) | 212 (12) | 129 (13) | 262 (13) | 152 (11) | 274 (13) | 101 (13) | 1,153 (12) | 998 (11) |
| ≥30 years | 144 (12) | 154 (10) | 201 (8) | 307 (7) | 139 (8) | 101 (10) | 195 (10) | 130 (10) | 209 (10) | 63 (8) | 888 (9) | 755 (9) |
| Sex^d^ |  |  |  |  |  |  |  |  |  |  |  |  |
| Female | 565 (45) | 733 (46) | 1,034 (43) | 1,817 (44) | 797 (43) | 425 (43) | 946 (46) | 587 (43) | 1,003 (46) | 325 (40) | 4,345 (45) | 3,887 (44) |
| Male | 677 (55) | 859 (54) | 1,345 (57) | 2,289 (56) | 1,039 (57) | 558 (57) | 1,093 (54) | 772 (57) | 1,163 (54) | 479 (60) | 5,317 (55) | 4,957 (56) |
| Gram stain |  |  |  |  |  |  |  |  |  |  |  |  |
| Done | 1,152 (93) | 1,356 (85) | 2,290 (96) | 3,336 (81) | 1,720 (94) | 740 (75) | 1,894 (93) | 1,055 (78) | 2,035 (94) | 651 (81) | 9,091 (94) | 7,138 (80) |
| BGN | 20 (2) | 33 (2) | 26 (1) | 52 (1) | 21 (1) | 10 (1) | 26 (1) | 21 (2) | 22 (1) | 12 (1) | 115 (1) | 128 (1) |
| BGP | 1 (0) | 9 (1) | 6 (0) | 20 (0) | 4 (0) | 9 (1) | 6 (0) | 13 (1) | 13 (1) | 12 (1) | 30 (0) | 63 (1) |
| DGN | 89 (7) | 108 (7) | 553 (23) | 838 (20) | 183 (10) | 65 (7) | 147 (7) | 104 (8) | 182 (8) | 63 (8) | 1,154 (12) | 1,178 (13) |
| DGP | 378 (30) | 264 (17) | 315 (13) | 363 (9) | 296 (16) | 128 (13) | 350 (17) | 138 (10) | 387 (18) | 102 (13) | 1,726 (18) | 995 (11) |
| Indeterminate | 0 (0) | 0 (0) | 0 (0) | 0 (0) | 0 (0) | 0 (0) | 0 (0) | 2 (0) | 2 (0) | 2 (0) | 2 (0) | 4 (0) |
| Negative | 664 (53) | 942 (59) | 1,390 (58) | 2,063 (50) | 1,216 (66) | 528 (53) | 1,365 (67) | 777 (57) | 1,429 (66) | 460 (57) | 6,064 (63) | 4,770 (54) |
| Not done | 90 (7) | 243 (15) | 89 (4) | 784 (19) | 116 (6) | 253 (25) | 145 (7) | 305 (22) | 131 (6) | 153 (19) | 571 (6) | 1,738 (20) |
| Probable cases^e^ |  |  |  |  |  |  |  |  |  |  |  |  |
| Yes | 800 (64) | 911 (57) | 1,459 (61) | 1,852 (45) | 953 (52) | 569 (57) | 1,029 (50) | 825 (61) | 1,119 (52) | 439 (54) | 5,360 (55) | 4,596 (52) |
| No | 442 (36) | 688 (43) | 920 (39) | 2,268 (55) | 883 (48) | 424 (43) | 1,010 (50) | 535 (39) | 1,047 (48) | 365 (46) | 4,302 (45) | 4,280 (48) |
| Reported deaths |  |  |  |  |  |  |  |  |  |  |  |  |
| Yes | 199 (16) | 220 (14) | 184 (8) | 406 (10) | 190 (10) | 145 (15) | 193 (9) | 156 (11) | 213 (10) | 90 (11) | 979 (10) | 1,017 (11) |
| No | 1,043 (84) | 1,379 (86) | 2,195 (92) | 3,714 (90) | 1,646 (90) | 848 (85) | 1,846 (91) | 1,204 (89) | 1,953 (90) | 714 (89) | 8,683 (90) | 7,859 (89) |

Abbreviations: BGN, Gram negative bacilli; BGP, Gram positive bacilli; CSF, cerebrospinal fluid; DGN, Gram negative diplococci; DGP, Gram positive diplococci.

^a^ A suspected meningitis case was defined as being tested at a national reference laboratory if it was tested by culture and/or real-time polymerase chain reaction.

^b^ Suspected bacterial meningitis is defined as sudden onset of fever ≥38.5°C with one of the following signs: neck stiffness, altered consciousness, or other meningeal signs (including flaccid neck, bulging fontanel, or convulsions in young children).

^c^ 68 cases missing age

^d^ 32 cases missing sex

^e^ Probable bacterial meningitis is a suspected case with turbid, cloudy, purulent, or xanthochromic CSF; or presence of DGN, DGP, or BGN on microscopic examination of

CSF; or a CSF white cell count >10/mm^3^.
